# Optimizing Cutting Parameters for Enhanced Control of Temperature, Cutting Forces, and Energy Consumption in Dry Turning of Ti6Al4V Alloy

**DOI:** 10.3390/ma18050942

**Published:** 2025-02-21

**Authors:** Manuel Herrera Fernández, Sergio Martín-Béjar, Lorenzo Sevilla Hurtado, Francisco Javier Trujillo Vilches

**Affiliations:** Department of Civil, Materials and Manufacturing Engineering, Engineering School, University of Malaga, 29071 Malaga, Spain; mherrera@uma.es (M.H.F.); lsevilla@uma.es (L.S.H.); trujillov@uma.es (F.J.T.V.)

**Keywords:** aeronautical industry, Ti6Al4V, lightweight alloys, machinability, dry machining, temperature, Energy consumption, mathematical models, optimization

## Abstract

This study aims to analyze the influence of cutting parameters (cutting speed, feed rate, and depth of cut) on cutting temperature, forces, and energy consumption during the dry turning of Ti6Al4V, providing an optimized machining strategy to improve efficiency and sustainability. Due to the challenges of machining this alloy, such as high temperatures and tool wear, response surface methodology (RSM) was used to develop second-degree polynomial models, and analysis of variance (ANOVA) identified the most influential factors. The results indicate that depth of cut has the highest impact on cutting temperature (42.59%), cutting forces (53.08%, 74.73%, and 48.87% in the respective force components), and power consumption (49.78%), while feed rate is the dominant factor in energy consumption (63.36%). Gray relational analysis (GRA) was applied to optimize machining conditions based on the developed models, allowing a wider selection of cutting parameters beyond the experimental values. These findings provide a valuable tool for the industry, offering manufacturers a data-driven approach to optimizing the machining of Ti6Al4V and reducing energy consumption and tool wear while improving process stability. The proposed methodology enhances sustainability and cost-efficiency in titanium alloy machining, particularly in the aeronautical sector.

## 1. Introduction

The aeronautical industry plays a vital role in modern society, not only by enabling global transportation but also by driving technological innovation. Due to the need to optimize performance while minimizing weight, the manufacture of aircraft relies heavily on lightweight alloys [1,2]. Among these materials, the Ti6Al4V alloy has become essential because of its excellent density-to-mechanical properties ratio. This alloy provides high strength, corrosion resistance, and durability, which are critical for aeronautical applications [3,4].

Ti6Al4V is widely employed in the manufacturing of structural and functional components of aircraft, such as engine parts, landing gear assemblies, and fuselage elements. These components must meet strict requirements regarding weight, strength, and safety. To ensure compliance with the high dimensional accuracy standards demanded by the aerospace industry, machining processes are extensively used, as they offer better control over the geometry of the final parts [5,6].

A notable trend in the aeronautical sector is the increasing adoption of dry machining operations. This approach aims to address environmental concerns by eliminating cutting fluids, which are toxic, difficult to recycle, and environmentally harmful [7]. However, the absence of lubricants makes dry machining more aggressive and poses significant challenges, particularly when hard materials like Ti6Al4V are machined [8,9,10].

The machinability of Ti6Al4V becomes a critical issue in this context. Machinability refers to how easily a material can be cut under specific conditions, influencing tool wear, surface finish, and overall process efficiency [11,12]. In dry conditions, the lack of cooling exacerbates issues like high temperatures, rapid tool wear, and surface defects, thereby negatively affecting machinability. As a result, the aerospace industry must carefully evaluate the behavior of this alloy to ensure an optimal balance between environmental sustainability and process efficiency [13,14].

Machinability is assessed through several variables that reflect the performance and efficiency of the machining process [15]. Among these, temperature [16,17], forces during the cutting process [18,19], and energy consumption [20,21] are particularly significant due to their practical relevance in machining operations. These variables are critical not only from a technical perspective but also because they directly or indirectly affect the economic viability of the process.

These variables are interconnected, and their impact on the production process extends beyond individual measurements. For instance, higher cutting forces and temperatures not only increase tool wear [16,22] but may also lead to product defects, resulting in scrap material and rework costs. In turn, frequent tool changes and rework translate into increased downtime and higher energy consumption. As a result, maintaining optimal values for these variables is essential to ensure the process efficiency, the process sustainability and the product conformity [23].

One way to mitigate the challenges associated with machining Ti6Al4V is through the optimization of cutting parameters, such as cutting speed, feed rate, and depth of cut. These variables have a direct impact on the machinability of the alloy and, therefore, must be thoroughly studied to achieve optimal process conditions. The optimal selection of these parameters allows manufacturers to minimize tool wear, control heat generation, and ensure dimensional precision, which are all essential for meeting the stringent requirements of the aerospace industry.

Given the complexity of machining operations and the various factors involved, it is crucial to develop mathematical models that can predict the behavior of key variables under different machining conditions. Such models help identify optimal parameter configurations, improve process stability, and reduce experimental costs. Ultimately, these predictive tools are essential for advancing knowledge-based manufacturing, enabling the industry to produce high-quality components while minimizing environmental impacts [24,25].

A variety of models are currently being developed, including polynomial, potential, and exponential models, among others. Additionally, the use of artificial intelligence is facilitating the creation of more complex models; however, its application usually requires a very large amount of data to arrive at satisfactory results, which entails a significant economic cost in terms of experimentation [26].

In the context of scientific research, it is customary to conduct analytical studies that encompass all variables pertinent to the process, thereby facilitating an evaluation of the physical behavior of the problem. In this regard, S. Velchev et al. [27] propose a mathematical model for the electrical energy consumed in a turning operation as a function of cutting speed (*v_c_*), feed (*f*), and the depth of cut (*a_p_*). In this case, the model is of the potential type, and the authors expose that the exponents depend on the characteristics of the material to which the model is to be applied. Additionally, they consider the value of the cutting time in the model, thereby generalizing it to any machining condition.

An alternative method for evaluating the process is through the generation of models using finite elements, which allows the process to be studied through simulation, thereby reducing the need for experimentation. In [28], a finite element model of the turning of an AISI 52100 steel is generated. This was used as the basis for a degree-2 polynomial model, created using the response surface methodology (RSM). This model relates the cutting temperature and the power consumed with the three cutting parameters (*v_c_*, *f*, and *a_p_*). The resulting models demonstrate a high degree of correlation, with R^2^ values of 0.8647 for temperature and 0.855 for power consumption.

In similar research, Tebassi et al. [29] employed RSM to construct a model for turning Inconel 718, encompassing surface roughness, forces during machining, the material removal rate (MRR), and power consumption as a function of the three cutting parameters (*v_c_*, *f*, and *a_p_*). The resulting models exhibited a high degree of correlation, with R^2^ values exceeding 0.94 in all instances.

In their study of the turning of hardened stainless steel AISI420, R. Nur et al. [20] varied the values of *v_c_* between 100 and 170 m/min and of *f* between 0.10 and 0.16 mm/rev, remaining constant *a_p_* (0.2 mm). They generated polynomial models for surface roughness, tool life, the resultant forces during machining, and the energy consumed. From these models, the optimal cutting conditions for machining within the range of values studied were identified, with values of 132.42 m/min and 0.12 mm/rev.

Similarly, in the case of the Ti6Al4V alloy, different works have also been developed with the objective of establishing a mathematical model, as in [30], in which an analytical model for the calculation of cutting forces in the milling of the Ti6Al4V alloy for different values of feed per tooth and depth of cut is presented.

In a previous study [31], Bai et al. developed a finite element simulation of an orthogonal cutting process of a Ti6Al4V alloy. The experimental results are compared with those obtained with the model, which considers cutting speeds from 20 to 140 m/min and feed rates of 0.05, 0.075, and 0.10 mm/rev. An analytical model is developed to relate the cutting process to the value of the cutting forces. The results demonstrate a high degree of scatter, particularly at low values of *f*, while the model results for *f* = 0.10 mm/rev exhibit a high degree of agreement with the experimental outcomes.

In a further study, J.O. Obiko et al. [32] developed a finite element model for the orthogonal cutting of the Ti6Al4V alloy. This model was used to investigate the influence of the three cutting parameters on temperature, cutting force, and power consumption. Once the model had been generated, the process was simulated for a range of values for the cutting speed, feed rate, and axial pitch. These values were selected to encompass the typical range of parameters used in the experimental procedure. The values used for the simulation were as follows: *v_c_* = 30–150 m/min, *f* = 0.10–0.30 mm/rev, and *a_p_* = 0.25–2.00 mm. This enables the generation of polynomial models of degree 1 for the three variables under study (*T*, *F_c_* and *P*), as a function of the three cutting parameters, with R^2^ values of 0.80, 0.97, and 0.90, respectively, being obtained.

In their study, Sun et al. [33] investigated the impact of cutting parameters on temperature during a Ti6Al4V milling operation. In his study, he considers a range of cutting speeds, feed rates, radial feeds, and axial feeds in order to obtain the values of cutting tool and chip temperature. From the experimental results, he develops a potential-type model to establish a relationship between temperature and cutting parameters.

M. Younas et al. [34] investigate the impact of cutting parameters (depth of cut, 0.01–4.5 mm; feed rate, 0.01–0.26 mm/rev; cutting speed, 45–180 m/min) on specific cutting energy, wear rates, surface roughness, and material removal rates in the dry turning of Ti6Al4V alloy. A gray relational analysis was carried out on the experimental results, which yielded a polynomic mathematical model of grade 2. This model relates the gray relational grade with the three cutting parameters under study and presents an excellent fit with R^2^ values of 0.968.

Following an assessment of the work completed thus far, it can be concluded that the RSM methodology, which is extensively employed in the investigation of the impact of cutting parameters on a range of variables associated with the machining process, enables the development of mathematical models with a high degree of accuracy. This methodology has been applied to the study of a variety of metallic alloys, including the Ti6Al4V alloy. Nevertheless, the studies conducted thus far have not encompassed a comprehensive range of cutting parameter values or considered all the cutting parameters inherent to a turning operation. It is important to acknowledge that previous research has established models that correlate cutting parameters with variables that assess the performance of the cutting process. However, these models have not been used for the purpose of optimizing the cutting operation.

Therefore, the objective of the present study is to examine the impact of cutting parameters (*v_c_*, *f*, and *a_p_*) on temperature, forces, and electrical energy consumption during a dry turning operation, with a particular focus on machinability impact. This has been achieved by utilizing a comprehensive range of experimental values for the cutting parameters in a dry turning operation involving Ti6Al4V alloy. Based on these results, a series of polynomial models, through the implementation of response surface methodology (RSM), have been constructed to establish a relationship between the cutting parameters and the variables under study. In addition, a normalized analysis of variance (ANOVA) was used to evaluate the influence of the cutting variables in the machinability variables. Finally, the models were employed in a gray relational analysis (GRA), which facilitated the optimization of the cutting process. This approach yields more accurate results than those obtained from solely considering the experimental results.

## 2. Materials and Methods

In the present study, a set of Ti6Al4V alloy specimens were turned under dry conditions. The tests were performed in a turning center, where the values of cutting speed (*v_c_*), feed (*f*), and depth of cut (*a_p_*) were controlled to assess their effect on the cutting temperature, cutting forces, and electrical energy consumption throughout the cutting process.

In order to comply with environmental regulations, the cutting operation was conducted without the use of cutting fluids, which are toxic and non-recyclable. This resulted in more aggressive machining conditions, which have limited the values of the cutting parameters that could be employed. In addition, the selected cutting parameters correspond to common industrial values for the dry machining of this alloy. Moreover, the chosen ranges include conditions representative of finishing and roughing operations, allowing for a comprehensive analysis of the machining process. Table 1 shows the values of the cutting parameters that were used in the machining operation.

The machining process was carried out using a rhombic insert with ISO reference DCGT 11 T3 08-MM YG401. This tool has an HC substrate and a PVD TiAlSiN coating recommended for the machining of titanium alloys. The tool was positioned at an angle of 93° to the main cutting-edge angle, which is suitable for the straight turning tests. It is important to highlight that modifying the geometric characteristics of the tool or the main cutting-edge angle would alter the machining conditions. Table 2 shows the geometrical characteristics of the cutting tool.

Online monitoring of the output variables under study (cutting temperature, cutting forces, and energy consumption) was conducted to record values while the machining operation was being carried out.

**Figure 1:**
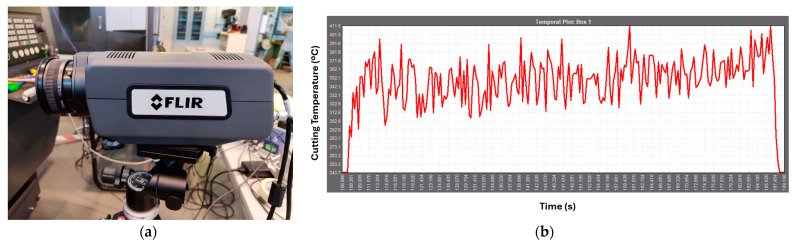
Cutting temperature acquisition: (**a**) thermographic camera; (**b**) temperature values along the machining process.

The temperature was recorded using a FLIR thermographic camera, model A6750 (Goleta, CA, USA). The thermographic images were treated with ResearchIR software (Version 4), obtaining the maximum temperature value in each frame analyzed. During the machining process, four frames per second were obtained, which generated a sufficient number of records to determine the value of the cutting temperature. In addition, the emissivity value considered for Ti6Al4V in this study is 0.5. This value was determined using the blackbody comparison method, following the guidelines of ISO 18434-1:2008 [35]. The temperature value was subsequently considered to be the average value obtained throughout the machining process (Figure 1).

**Figure 2:**
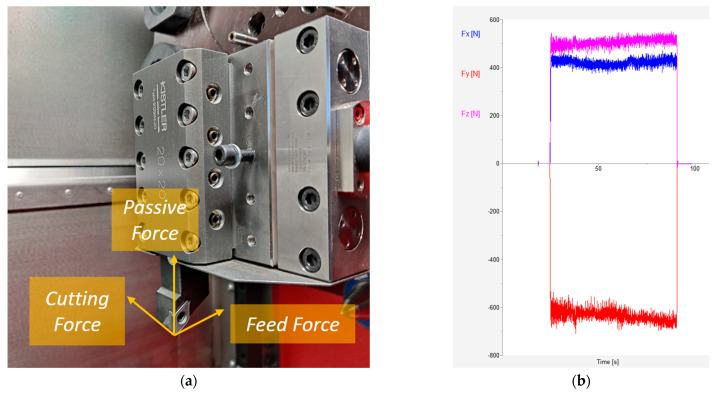
Forces measurement during the cutting process. (**a**) Dynamometer; (**b**) forces signal acquisition.

To obtain the forces during the cutting process, a Kitsler 9129-A dynamometer (Winterthur, ZH, CH), with an acquisition software denominated Dynoware (Version 2.6), was utilized (Figure 2). The signals acquired correspond with the forces values in three directions: passive force (*F_x_*), cutting force (*F_y_*), and feed force (*F_z_*). The average value of the entire machining operation was considered. The system enables the generation of force records with a frequency of 100 Hz. Based on these results, the value of the resultant force (*R*) was calculated.

**Figure 3:**
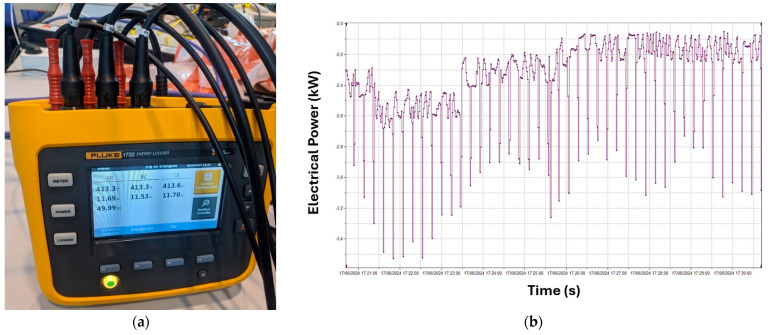
Electric power and energy consumption measurement. (**a**) Network analyzer equipment; (**b**) electrical signal acquired.

Subsequently, throughout the online monitoring procedure, the active power and active energy consumption during the machining operation were measured using a network analyzer FLUKE, model 1732 (Everett, WA, USA) (Figure 3). The system allows the registration of one instance per second. In the case of active power, the mean value of the entire machining operation has been considered, while the active energy consumption is represented by the sum of each record obtained during the cutting operation.

The experimental results yielded the generation of degree-2 polynomial models for each of the variables under study, as a function of the three cutting parameters employed during the machining process (*v_c_*, *f*, and *a_p_*), using response surface methodology (RSM) techniques. This also makes it possible to evaluate the influence of each parameter on the temperature, forces, and energy consumption through the use of a normalized analysis of variance (ANOVA). Finally, a gray relational analysis (GRA) was conducted in order to identify the optimal cutting parameters for dry machining of the alloy, with the objective of achieving the lowest values for temperature, forces, and energy consumption.

## 3. Results

The experimental methodology yielded a series of results of the cutting temperature, the forces generated during the machining process, and the electrical power and energy consumption associated with the removal of material. The results, obtained through online monitoring, are presented below and discussed in relation to the impact of cutting speed, feed, and depth of cut variables on each cutting parameter combination. Additionally, various models derived from RSM are presented, which establish a relationship between the variables under investigation and the cutting parameters. Moreover, the methodology allows for the determination of the degree of significance of each of the cutting parameters on the variables under study through a standardized analysis of variance (ANOVA).

Ultimately, the aim is to determine the optimal machining conditions, taking into account all the experimental results obtained through a gray relational analysis (GRA).

### 3.1. Cutting Temperature Analysis

**Figure 4:**
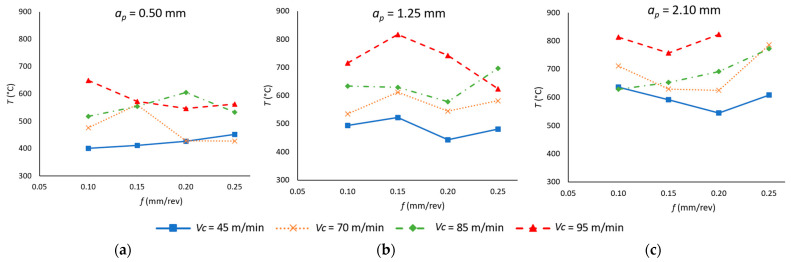
Mean temperature values in the cutting process *T* (°C) as a function of the feed rate (*f*) and cutting speed (*v_c_*) for each depth of cut (*a_p_*): (**a**) *a_p_* = 0.50 mm; (**b**) *a_p_* = 1.25 mm; (**c**) *a_p_* = 2.10 mm.

Figure 4 shows the temperature evolution during the cutting process as a function of *v_c_*, *f*, and *a_p_* that have been studied in the context of the machining operation. As the temperature values have been obtained at several points throughout the machining operation, the values displayed represent the mean of the entire process.

As illustrated in Figure 4, an increase in both *v_c_* and *a_p_* appears to result in higher temperature values during the machining process. Regardless of the values of *v_c_* and *a_p_*, there is no discernible trend in temperature for the variable *f*. These behaviors are associated with the effect that the cutting parameters have on material removal. Higher *v_c_* values increase the chip friction with the rake face of the tool, while an increase in *a_p_* means greater material removal, generating a chip with a larger cross-section and, therefore, an increase in the contact surface of the chip with the cutting tool, increasing the friction between chip and tool [36,37]. Nevertheless, although higher values of *f* also result in a larger cross-section chip, this parameter generates a higher chip evacuation speed, thereby facilitating the dissipation of the heat generated in the cutting process. Consequently, its effect on temperature is less significant.

It can be observed that, for low values of *a_p_* (Figure 4a), the influence of cutting speed is more relevant at low feed rates (0.10 mm/rev), where an increase of 62% has been observed. In contrast, at high feed rates (0.20 mm/rev), with a variation of 24%, this effect is reduced. This is due to an increase in chip evacuation at high values of *f*, which generates an increase in heat dissipation, thereby reducing the effect of *v_c_* on temperature. However, for higher values of *a_p_* (Figure 4c), the effect of *f* on temperature by varying *v_c_* is less relevant due to the larger increase in chip cross-section associated with higher *f* and *a_p_*, which compensates for the increase in heat dissipation due to a higher value of *f*.

In order to evaluate the degree of significance of each cutting parameter, a normalized analysis of variance (ANOVA) was carried out. The results are shown in Table 3.

The ANOVA results highlight that *a_p_* and *v_c_* are the most significant factors influencing cutting temperature, with contributions of 42.59% and 40.74%, respectively. This confirms that these parameters are the primary drivers of heat generation in the machining process, as higher cutting speeds lead to increased friction and thermal loads, while greater depths of cut result in larger contact areas and higher energy input into the material.

In contrast, the feed rate (*f*) exhibits a negligible influence on temperature variation, contributing only 0.01%, which suggests that its effect is overshadowed by the dominant role of *v_c_* and *a_p_*. Additionally, quadratic terms and interaction effects have relatively minor contributions, indicating that the temperature response is primarily governed by the linear effects of the main cutting parameters.

In Equation (1), a second-order polynomial model is presented that relates the cutting temperature to the various cutting parameters employed during the experimental phase. The model demonstrates a reasonable fit (R^2^ = 0.8236), indicating its suitability for predicting temperature within the specified range of values for *v_c_*, *f*, and *a_p_*.

*T* (°C) = 225 + 1.09 *v_c_* − 297 *f* + 180.1 *a_p_* + 0.0129 *v_c_*^2^ + 1197 *f*^2^ − 34.3 *a_p_*^2^ − 4.13 *v_c_* · *f* − 0.094 *v_c_* · *a_p_* + 74 *f* · *a_p_* (1)

In comparison with other research, E. Abdelnasser et al. [38] present a degree-two polynomial model relating temperature to the cutting parameters (*v_c_*, *f*, *a_p_*), which presents an R^2^ of 0.952. Nevertheless, the present study was conducted for exceptionally low values of *a_p_* (0.15–0.25 mm), while the values of *f* (0.10–0.20 mm/rev) and *v_c_* (50–150 m/min) were maintained. A reduction in the value of *a_p_* has the effect of reducing vibration and enhancing process stability, which in turn leads to a reduction in experimental variability, particularly in view of the pivotal role played by *a_p_* in determining cutting temperature. Consequently, the presented model in this work not only exhibits a favorable fit but also encompasses a broader range of cutting parameters than those previously documented in the literature.

### 3.2. Forces Analysis in the Cutting Operation

**Figure 5:**
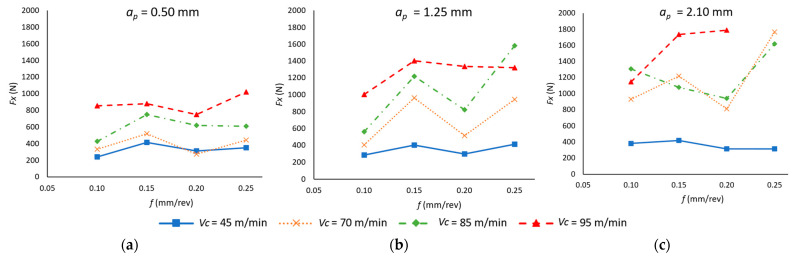
Mean passive forces values in the cutting process as a function of the feed rate (*f*) and cutting speed (*v_c_*) for each depth of cut (*a_p_*): (**a**) *a_p_* = 0.50 mm; (**b**) *a_p_* = 1.25 mm; (**c**) *a_p_* = 2.10 mm.

Figure 5 illustrates the experimental values of the passive forces (*F_x_*) across the full range of *v_c_*, *f*, and *a_p_* values that were tested. Firstly, with regard to the impact of velocity on *F_x_*, it can be observed that, for all the tested values of *a_p_*, an increase in *v_c_* tends to elevate the value of *F_x_*, with this increase being more pronounced at elevated *a_p_* values. Indeed, an increase in *a_p_* also tends to elevate the value of *F_x_*, with the highest force being observed for the highest value of *v_c_* (95 m/min) and *a_p_* (2.1 mm). Conversely, with respect to *f*, there is also an increase in *F_x_* as its value increases. However, this behavior is not as clear as with *v_c_* and *a_p_* since, for intermediate values of *f* (0.15–0.20 mm/rev), there are variations in its tendency.

This behavior is consistent with the results obtained from the ANOVA (Table 4), which determined that *v_c_*, *a_p_*, and *f* are statistically significant variables in the values obtained for *F_x_*. In this case, *v_c_* is the most influential parameter, accounting for 53.08% of the total variability and highlighting its dominant role in force generation. The cutting parameter *a_p_* also has a considerable impact, contributing 19.81%, confirming its direct influence on cutting forces due to the increased contact area between the tool and the material. Conversely, *f* exhibits the lowest impact among the main factors, with a contribution of only 4.16%, indicating that variations in feed have a comparatively smaller effect on *F_x_*.

Additionally, the interaction between *v_c_* and *a_p_* is also statistically significant, contributing 5.47%, suggesting that these two parameters do not act independently but rather have a combined influence on force generation. The quadratic terms and other interactions show minimal impact, reinforcing that the main effects of *v_c_* and *a_p_* govern the behavior of *F_x_*. These findings further support the notion that optimizing cutting speed and depth of cut is crucial for controlling cutting forces in the machining of Ti6Al4V.

The experimental results have been used to generate a polynomial model of degree 2 (Equation (2)), which allows the value of *F_x_* to be predicted as a function of the cutting parameters. The model demonstrates a reasonable correlation (R^2^ = 0.806).

*F_x_* (N) = 1062 − 18.3 *v_c_* − 4682 *f* − 394 *a_p_* + 0.115 *v_c_*
^2^ + 3732 *f*^2^ − 47 *a_p_*^2^ + 49.6 *v_c_* · *f* + 8.79 *v_c_* · *a_p_* + 1232 *f* · *a_p_* (2)

**Figure 6:**
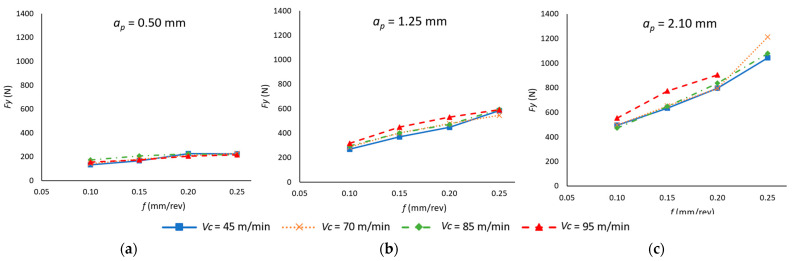
Mean cutting forces’ values in the cutting process as a function of the feed rate (*f*) and cutting speed (*v_c_*) for each depth of cut (*a_p_*): (**a**) *a_p_* = 0.50 mm; (**b**) *a_p_* = 1.25 mm; (**c**) *a_p_* = 2.10 mm.

With regard to the value of the cutting force (*F_y_*), the resulting data from the machining operations are presented in Figure 6. As was the case for *F_x_*, an increase in the cutting values tends to result in an increase in the value of the cutting force. Nevertheless, in this instance, an increase in *f* shows a more pronounced influence on *F_y_* than on *F_x_*. Indeed, for high values of *f* and *a_p_*, an increase in *f* from 0.10 mm/rev to 0.25 mm/rev has been observed to result in a 250% increase. Additionally, *a_p_* plays a significant role in the behavior of *F_y_*. At elevated values of *f* (0.25 mm/rev), the rise in *a_p_* has resulted in a 533% increase in *F_y_*. In this instance, *v_c_* is not as influential as *f* and *a_p_*. Nevertheless, an increase in *v_c_* tends to yield higher *F_y_* values, with this phenomenon being more pronounced at elevated values of *a_p_* (2.1 mm).

The results of the ANOVA (Table 5) indicate that, as in *F_x_*, the three cutting parameters are statistically significant variables. However, their level of influence differs, with *a_p_* being the most dominant factor (74.73%), while *v_c_* has the lowest impact (0.35%). This reinforces the idea that *a_p_* plays a crucial role in force generation, whereas *v_c_* has a negligible effect. Additionally, as was previously observed in the analysis of the experimental outcomes, the interaction between *f* and *a_p_* is also statistically significant (8.13%), highlighting their combined effect on force distribution.

Furthermore, the ANOVA results reveal a remarkably low error percentage (1.53%), indicating that the selected model effectively captures the variability in *F_y_* and that the analyzed cutting parameters sufficiently explain the observed trends.

Equation (3) illustrates the model that correlates *F_y_* with the aforementioned cutting parameters. In this instance, the model demonstrates an exceptionally strong fit (R^2^ = 0.985).

*F_y_* (N) = 269 − 1.69 *v_c_* − 1159 *f* − 187 *a_p_* + 0.015 *v_c_*
^2^ + 2060 *f*^2^ + 44.7 *a_p_*^2^ − 3.29 *v_c_* · *f +* 0.797 *v_c_* · *a_p_* + 2172 *f* · *a_p_* (3)

**Figure 7:**
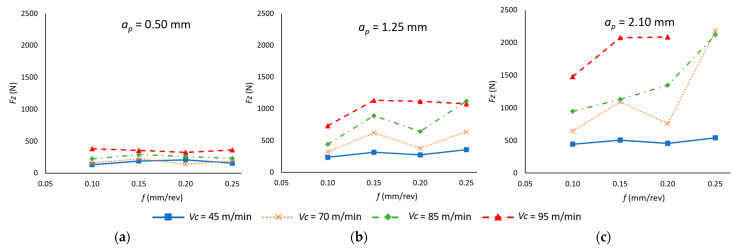
Mean feed forces’ values in the cutting process as a function of the feed rate (*f*) and cutting speed (*v_c_*), for each depth of cut (*a_p_*): (**a**) *a_p_* = 0.50 mm; (**b**) *a_p_* = 1.25 mm; (**c**) *a_p_* = 2.10 mm.

Figure 7 shows the mean value of the feed force (*F_z_*) as a function of the cutting parameters. An increase in *v_c_*, *f*, and *a_p_* leads to a rise in *F_z_*, consistent with the trends observed for *F_x_* and *F_y_*. However, the impact of each parameter differs in magnitude. The results of the ANOVA (Table 6) confirm that *a_p_* is the most influential factor (48.87%), followed by *v_c_* (30.23%), while *f* has a notably lower impact (5.87%). Additionally, the interaction between *v_c_* and *a_p_* is statistically significant (12.48%), reinforcing the strong combined effect of these parameters on feed force generation.

Furthermore, the influence of *v_c_* on *F_z_* becomes more pronounced as *a_p_* increases, while *f* remains the least influential. This trend aligns with the role of *a_p_* in determining the contact area between the tool and the material. The following analysis further explores how the dominance of *F_z_* depends on the magnitude of *a_p_*, particularly in relation to *F_x_*.

For values of *a_p_* greater than 2.1 mm, *F_z_* is the force with the highest value. Conversely, for values of *a_p_* between 0.5 and 1.25 mm, *F_x_* has the highest significance in the forces applied for the removal of the material. This is due to the fact that at higher *a_p_* values, in the feed direction (*F_z_*), the tool faces a larger section of material to be machined, which causes a significant increase in the force in this direction. However, *F_x_*, which corresponds to the reaction of the material on the surface that has just been turned, although also affected by the value of *a_p_*, the fact that the specimen to be machined undergoes a bending deformation tends to reduce the effect of *a_p_*.

As with the preceding forces, a degree-two polynomial model is presented (Equation (4)) that correlates the value of *F_z_* with the values of the cutting parameters. In this case, the model demonstrates a higher fit (R^2^ = 0.895) compared to *F_x_*. Despite observing a comparable influence of the cutting parameters on *F_z_*, the experimental results exhibited enhanced stability when *v_c_*, *f* and *a_p_* were varied.

*F_z_* (N) = 2484 − 43.4 *v_c_* − 7558 *f* − 1421 *a_p_* + 0.218 *v_c_*
^2^ + 5707 *f*^2^ + 118.2 *a_p_*^2^ + 48.8 *v_c_* · *f* + 15.54 *v_c_* · *a_p_* + 3467 *f* · *a_p_* (4)

**Figure 8:**
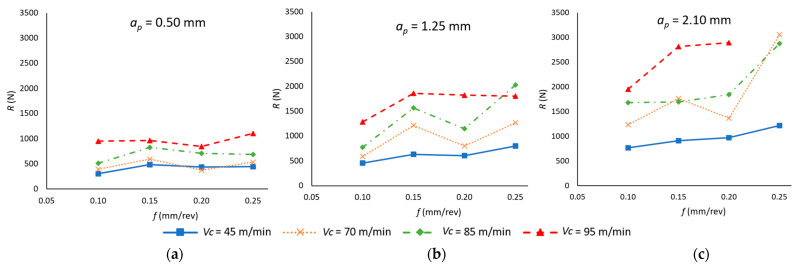
Cutting forces resultant as a function of the feed rate (*f*) and cutting speed (*v_c_*) for each depth of cut (*a_p_*): (**a**) *a_p_* = 0.50 mm; (**b**) *a_p_* = 1.25 mm; (**c**) *a_p_* = 2.10 mm.

Finally, the total value of the resultant force of the three forces was obtained (Equation (5)). These values were obtained for each cutting parameter combinations, and their results are shown in Figure 8.

(5) $$R=\sqrt{F_{x}^{2}+F_{y}^{2}+F_{z}^{2}}$$

With regard to the impact of cutting parameters on R, a comparable trend to that observed for *F_x_* and *F_z_* is evident due to the fact that they are the forces with the highest magnitude, in contrast to *F_y_*, which has a lower value. However, the greater significance of *f* that has been observed in *F_y_* compared to its lesser influence on *F_x_* and *F_z_* is also represented in the value of R, especially for low values of *v_c_* (45 m/min). This is particularly evident as a more significant increase in R is observed when *f* increases, especially at higher values of *a_p_* (1.25–2.10 mm). Furthermore, the greater relevance of *F_x_* and *F_z_* on R can be observed as the influence of *v_c_* at higher values of *a_p_* is more relevant, as was the case for each of these variables analyzed independently.

This behavior is reflected in the ANOVA (Table 7), which confirms that the three cutting parameters are statistically significant in determining the resultant force (*R*). Among them, *a_p_* has the highest influence (43.35%), followed by *v_c_* (32.24%), while *f* has a notably lower impact (8.14%). These results are consistent with the trends observed for *F_x_* and *F_z_*, reinforcing the dominant role of *a_p_* and *v_c_* in force generation.

Moreover, the interaction between *v_c_* and *a_p_* is also statistically significant (5.64%), highlighting their combined effect on *R*, as previously observed in *F_x_* and *F_z_*. However, in this case, the correlation between *f* and *a_p_* also plays a relevant role (4.10%), influenced by the contribution of *F_y_*, suggesting that the feed rate, while less dominant, still affects the resultant force through its interaction with *a_p_*.

In good agreement with the predictive performance of the polynomial models for each of the forces, Equation (6) presents the functional relationship between R and the cutting parameters evaluated. In this instance, a similarly favorable outcome is achieved (R^2^ = 0.888), with a value situated between those obtained for the preceding models.

*R* (N) = 2398 − 43.3 *v_c_* − 7655 *f* − 1214 *a_p_* + 0.273 *v_c_*
^2^ + 7407 *f*^2^ + 97 *a_p_*^2^ + 50 *v_c_* · *f* + 14.04 *v_c_* · *a_p_* + 4079 *f* · *a_p_* (6)

The results obtained for each of the forces demonstrate that the influence exerted by *a_p_* on the values obtained should be highlighted. It is important to note that the range of values studied for *a_p_* (0.50–2.10 mm) is quite wide, which amplifies its influence on the forces. Additionally, an increase in *a_p_* signifies a rise in the quantity of material removed during the cutting process, utilizing a more extensive cutting edge. Consequently, greater efforts are required.

The quantity of material removed during the cutting process is also contingent upon the value of *f*; thus, an increase in its value results in elevated forces during the machining process. Nevertheless, values of *f* between 0.10 and 0.25 mm/rev have been utilized, thereby rendering the increase in material removed in the cutting process relatively insignificant when compared to the range of values employed for *a_p_* (0.50 to 2.10 mm). However, in the values obtained for *F_x_*, the relevance of *f* is more significant than for *F_x_* and *F_z_*, due to the fact that the material being machined exerts a force perpendicular to the tool face, which coincides with the direction of *F_y_*.

With regard to the cutting speed, the range of values employed is considerable for the machining of this particular alloy (40–95 m/min), which has a detrimental impact on the increase in the values of the forces in all directions. Despite the influence of *v_c_* on the cutting temperature, its increase has not facilitated the removal of material due to the low thermal conductivity of titanium in comparison with other materials [39,40,41].

Consequently, the cutting parameters have a complex relationship with the cutting forces in turning, and the proper adjustment of these parameters is crucial to ensure an efficient and stable turning process.

The behavior of the cutting forces observed aligns with research [42,43,44], where the material removal process generates forces that depend on the interaction between the cutting tool and the workpiece. The results confirm that depth of cut plays a dominant role, as expected from classical machining theory, where an increase in this parameter leads to higher cutting forces due to the larger undeformed chip cross-section. Cutting speed also contributes significantly, influencing the strain rate and frictional conditions at the tool-chip interface, while feed rate primarily affects *F_y_*, in line with its direct relationship to chip thickness.

Understanding the distribution and magnitude of forces in the machining process is crucial for optimizing machining parameters to enhance process efficiency and tool life. Controlling these forces is particularly relevant in industry, where excessive mechanical loads can lead to tool wear, geometrical deviations, and unwanted vibrations that compromise part quality.

### 3.3. Electric Power and Energy Consumption Analysis in the Cutting Process

**Figure 9:**
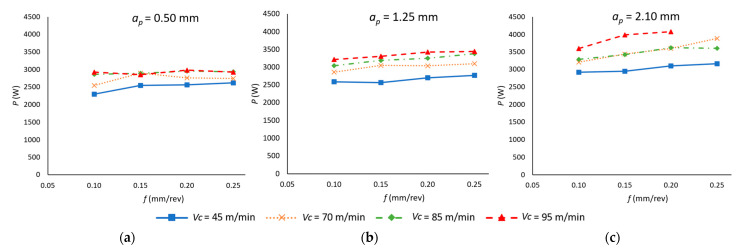
Active power in the cutting process as a function of the feed rate (*f*) and cutting speed (*v_c_*) for each depth of cut (*a_p_*): (**a**) *a_p_* = 0.50 mm; (**b**) *a_p_* = 1.25 mm; (**c**) *a_p_* = 2.10 mm.

Figure 9 shows the values of the active power (*P*) of the machining center during the cutting operation as a function of the varying values of *v_c_*, *f* and *a_p_*. It can be observed that an increase in each of the cutting parameters results in a corresponding increase in the value of the electrical power. This phenomenon can be attributed to two primary factors. Firstly, as the chip section increases due to an elevated *a_p_* and *f*, it becomes necessary to augment the mechanical torque in order to facilitate the cutting process. Secondly, it is essential to consider that higher *v_c_* values necessitate an analogous increase in the spindle rotation speed, with an increase in *P* being indispensable to enable its execution.

The ANOVA results (Table 8) confirm the statistical significance of *v_c_*, *f*, and *a_p_* in determining *P*. However, their influence is not uniform. The contribution of *a_p_* (49.78%) is the most dominant, reinforcing its role as the primary factor affecting power consumption. This is followed by *v_c_* (35.79%), which also plays a crucial role, while *f* (6.22%) exhibits the least impact.

A key aspect of these findings is the strong dependence of *P* on both *a_p_* and *v_c_*, which leads to a statistically significant interaction (*v_c_* · ap: 1.56%). This suggests that increasing either parameter alone does not fully capture their combined effect on power consumption. Instead, their simultaneous variation should be considered to better understand and optimize energy efficiency in the machining process.

While the influence of *f* is comparatively minor, its interaction with *a_p_* (1.04%) also presents a measurable effect, indicating that even small adjustments in the feed rate may have an impact when combined with variations in the depth of cut.

A second polynomial model of degree 2 was generated from the experimental data (Equation (7)), demonstrating a high level of correlation (R^2^ = 0.941).

*P* (W) = 1607 + 10.25 *v_c_* + 4329 *f* − 247 *a_p_* − 0.0139 *v_c_*
^2^ − 10457 *f*^2^ + 76.4 *a_p_*^2^ − 4.8 *v_c_* · *f* + 4.07 *v_c_* · *a_p_* + 1131 *f* · *a_p_* (7)

**Figure 10:**
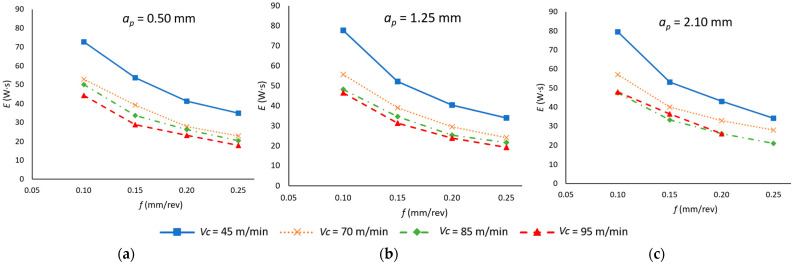
Active energy in the cutting process as a function of the feed rate (*f*) and cutting speed (*v_c_*) for each depth of cut (*a_p_*): (**a**) *a_p_* = 0.50 mm; (**b**) *a_p_* = 1.25 mm; (**c**) *a_p_* = 2.50 mm.

Furthermore, the active energy consumption (*E*) during the machining operation was quantified. The results are presented in Figure 10 as a function of the cutting parameters. As can be observed in this instance, an increase in both *f* and *v_c_* appears to result in a reduction in the energy consumed. This behavior is more pronounced in the case of *f*, where a reduction of up to 50% is observed when machining with low values of *v_c_*.

The increase in active power observed previously is offset by a reduction in energy consumption due to the influence of a higher value of *v_c_* and *f* on machining time, which is a more significant factor than the increase in electrical power.

The depth of cut is the parameter with the least relevance for energy consumption, given that it does not have a noticeable effect on machining time in a single machining operation. However, the increase in the active power necessary to remove a greater amount of material, associated with the increase in mechanical torque, means that the increase in *a_p_* tends to increase the value of *E*.

The results of the ANOVA (Table 9) indicate that all cutting parameters are statistically significant, highlighting their relevance in the machining operation. However, their influence varies considerably. Among them, *f* has the highest impact (63.36%), followed by *v_c_* (26.95%), while *a_p_* plays a minimal role (0.40%).

A notable aspect of these findings is the statistically significant correlation between *v_c_* and *f*, which can be attributed to their dominant influence on machining time. This relationship also explains why the quadratic components of *v_c_* (1.09%) and *f* (3.85%) remain relevant, reinforcing the need to analyze these parameters together when optimizing energy consumption in machining operations.

Ultimately, a model was developed (Equation (8)) that correlates the active energy consumed with the cutting parameters *v_c_*, *f*, and *a_p_*. This model demonstrates a high degree of fit (R^2^ = 0.987), indicating a high level of reliability in predicting the energy consumed during the cutting operation.

*E* (W·s) = 190.26 − 1.612 *v_c_* (m/min) − 769.3 *f* − 0.86 *a_p_* + 0.00582 *v_c_*
^2^ + 1165 *f*^2^ + 0.621 *a_p_*^2^ + 2.093 *v_c_* · *f* + 0.0119 *v_c_* · *a_p_* − 0.73 *f* · *a_p_* (8)

It is important to note that, regardless of the analysis carried out for a turning operation, when it is necessary to reduce the material to a depth of 2 mm, four operations of 0.5 mm are required. Consequently, the influence of *a_p_* would be more notable in this case, as at least four times the energy value obtained in an operation of *a_p_* = 0.5 mm would be necessary. This aspect is crucial to consider when analyzing machining times and production costs, as it not only multiplies machining times by four but also requires the inclusion of non-productive times, which correspond to the time required for the tool to move between machining operations when a shallower depth of cut is considered.

The trends observed in active power and energy consumption are consistent with machining research [45,46,47], where power demand is directly influenced by the forces acting on the tool and the cutting speed. Given that forces are largely governed by *a_p_*, it follows that this parameter also plays a significant role in determining power consumption. Additionally, the relationship between *v_c_* and *P* aligns with theoretical models, where an increase in speed raises the mechanical energy required for material removal, although with varying efficiency, depending on tool-workpiece interactions. The feed rate, while contributing less significantly, still influences the total energy expenditure due to its effect on cutting time.

From a manufacturing perspective, optimizing power and energy consumption is essential for improving process efficiency and reducing operational costs. The insights from this study are particularly relevant for industries working with Ti6Al4V, where controlling energy usage is crucial to maintaining sustainable machining practices. By selecting appropriate cutting parameters, manufacturers can minimize unnecessary power consumption, extend tool life, and reduce the environmental impact of machining operations, aligning with the growing demand for energy-efficient manufacturing strategies.

### 3.4. Gray Relational Analysis for the Machinability Variables

A gray relational analysis was conducted based on the experimental results obtained, taking into account all the variables that were the subject of study during the machining process. In this case, it is necessary to consider that, for the optimization of the problem, all the variables have the lowest possible value. This is so because higher values of temperature and forces would have a negative effect on tool wear. Additionally, electrical power could be a limiting value for a numerical control machine tool. Furthermore, a high consumption of active energy would mean a higher cost in the machining operation. In Table 10, the GRC value obtained for all the cutting combinations studied can be observed.

The results demonstrate that low values of the cutting parameters (*v_c_* = 40 m/min, *f* = 0.05 mm, and *a_p_* = 0.5 mm) exhibit optimal behavior across all studied variables despite being associated with the highest energy consumption. Nevertheless, the significance of energy consumption becomes evident in the subsequent conditions, wherein a second-best cutting condition is attained for *v_c_* = 70 m/min, *f* = 0.20 mm/rev, and *a_p_* = 0.5 mm. This illustrates the pivotal role of elevated machining speeds (*v_c_* and *f*) in optimizing the cutting process. Indeed, the resulting combinations demonstrate a range of feed rates between 0.20 and 0.25 mm/rev and a range of cutting speeds between 45 and 70 m/min.

In all cases, the most appropriate values are considered to be for a value of *a_p_* = 0.5 mm, which may be related to the high influence of this parameter on the temperature and forces analyzed.

Given that the models proposed for each variable demonstrate a satisfactory level of fit, a prediction was made for each variable, with variations of 1 m/min for *v_c_*, 0.01 mm/rev for *f*, and 0.01 mm for *a_p_*, within the range of cutting parameter values studied.

A new GRA analysis was conducted using the aforementioned values, the results of which are presented in Table 11. This table displays the ten best combinations of cutting parameters. The results demonstrate that the optimal conditions were achieved for the lowest *a_p_* (0.5 mm), while *v_c_* is within the low value range, and *f* is within the high value range.

As previously considered, energy consumption was obtained for a single turning operation without taking into account that machining a depth of cut of 2 mm would require four times the energy consumed in a cutting operation with an *a_p_* of 0.5 mm. Therefore, within the energy consumption of a single turning operation, the energy consumed in a cutting operation with an *a_p_* of 0.5 mm would be four times the energy consumed in a cutting operation with an *a_p_* of 2.1 mm. Accordingly, a proportionality coefficient (*n_i_*) was derived (Equation (9)) to amplify the value of the energy consumed as a function of the maximum value of *a_p_* considered. This allows the corrected value of the active energy consumed (*E*’) to be obtained for each value of *a_p_* (Equation (10)):

(9) $$n_{i}=\frac{2.1}{a_{p\;i}}$$

(10) $$E_{i}^{\prime }=n_{i}\cdot E_{i}$$

In previous analyses, when all variables in the GRA were considered, the forces were found to be of greater relevance in the optimization of the problem due to the fact that there were four variables compared to the temperature or electrical analysis. It thus appears reasonable to conduct the study on the basis of a single variable for the forces, with *R* representing the variable encompassing the forces in the three orthogonal directions. Moreover, the electrical power necessary to initiate the material can be regarded as a limiting factor in the selection of the machine tool, without affecting the characteristics of the resulting part. In consideration of these conditions and the satisfactory alignment of the proposed models, a GRA analysis was conducted on *T*, *R*, and the corrected value of active power consumption (*E*’), the results of which are presented in Table 12.

In this instance, the outcome is analogous to that of the preceding analysis, with elevated values of *f* exhibiting a greater influence, attributable to the abbreviated machining time and consequently reduced energy consumption. Conversely, low values of *v_c_* and *a_p_* are evident to mitigate the impact of temperature and forces during the specimen’s machining.

## 4. Conclusions

This work provides valuable insights into the impact of key machining parameters (*v_c_*, *f*, and *a_p_*), on critical variables such as cutting temperature, forces, and energy consumption. The study emphasizes the intricate interrelationship between these parameters and underscores their significance in achieving optimal machining conditions.

With regard to cutting temperature, the study illustrates that elevated values of *v_c_* and *a_p_* result in elevated temperatures due to intensified friction and greater material removal. However, the feed rate does not have a significant impact on temperature, as higher feed rates facilitate heat dissipation through efficient chip evacuation. This trend is corroborated by the statistical results obtained from ANOVA, which indicate that ap and *v_c_* are the most influential variables affecting temperature, with *a_p_* playing a more substantial role.

The force analysis serves to corroborate the significant impact of *a_p_* on the passive (*F_x_*), cutting (*F_y_*), and feed forces (*F_z_*) in question. For each force, an increase in *a_p_* or *f* resulted in elevated force values, while the effect of *v_c_* varied based on its interaction with other parameters. The degree-two polynomial models developed for each force demonstrated strong correlations, indicating their reliability for predicting force values within the studied range of parameters. The ANOVA results serve to reinforce the relevance of ap in determining forces, particularly in terms of its influence on the directionality and magnitude of each force.

In terms of power and energy consumption, the findings demonstrate a direct correlation between increases in *v_c_*, *f*, and *a_p_* and active power consumption. It is noteworthy that there is an inverse relationship between energy consumption and feed rate and cutting speed. This is primarily due to the reduction in machining time, which offsets the rise in power. These findings indicate that optimizing the aforementioned parameters is of paramount importance for reducing energy expenditure while ensuring effective material removal.

The gray relational analysis (GRA) conducted to determine optimal machining conditions identified that lower values of *v_c_* and *a_p_*, combined with higher feed rates, are advantageous in reducing temperature, forces, and energy consumption. The results demonstrate that an *a_p_* value of 0.5 mm consistently emerges as the optimal value, in alignment with the observed high influence of this parameter on the studied variables. Furthermore, the introduction of a proportionality coefficient (*n_i_*) in the calculation of energy consumption highlights the necessity of considering multiple operations in scenarios with higher *a_p_* values.

The research findings confirm the significance of the depth of cut (*a_p_*) as the most influential parameter in the machining process. The statistical models and GRA results demonstrate that the proper adjustment of these parameters is essential for balancing the trade-offs between temperature, forces, and energy consumption, thereby enhancing process stability and efficiency. This comprehensive analysis provides valuable guidance for optimizing turning operations and improving machinability in the context of complex alloys like titanium. Therefore, manufacturers can leverage these findings to implement more energy-efficient and cost-effective machining strategies, minimizing tool wear and maximizing process stability, while maintaining a focus on sustainability in manufacturing.

Due to the complexity of Ti6Al4V machining and its sensitivity to process parameters, further studies are needed to optimize cutting conditions and improve component performance. Future research could explore the effects of alternative cooling strategies, such as MQL or cryogenic cooling, on machinability variables. Additionally, this approach can be extended to the study of surface integrity, which is related to multiple variables and plays a crucial role in the performance of Ti6Al4V components. Moreover, the geometry of the cutting tool, including the main cutting-edge angle, significantly influences the machining process and should be further investigated to enhance tool performance and process efficiency.

## Figures and Tables

**Table 1:** Cutting parameters applied to the dry turning operation.

*v_c_* (m/min)	*f* (mm/rev)	*a_p_* (mm)
45	0.10	0.50
70	0.15	1.25
85	0.20	2.10
95	0.25	

**Table 2:** Cutting parameter applied in the dry turning operation.

Parameter	Value	Parameter	Value
Tool clearance angle	7°	Nose radius	0.80 mm
Tool rake angle	17°	Main cutting edge	93°
Tool included angle	55°	Cutting-edge length	10.83 mm

**Table 3:** ANOVA results for cutting parameters’ influence on the cutting temperature.

Source	DF	Adj SS	Adj MS	F Value	*p* Value	Impact (%)
Regression model	9	489,671	54,408	19.19	0.000	
*v_c_*	1	242,262	242,262	85.45	0.000	40.74
*f*	1	53	53	0.02	0.892	0.01
*a_p_*	1	253,159	253,159	89.29	0.000	42.59
*v_c_* ^2^	1	8499	8499	3.00	0.092	1.43
*f* ^2^	1	1179	1179	0.42	0.523	0.20
*a_p_* ^2^	1	824	824	0.29	0.593	0.14
*v_c_* · *f*	1	40	40	0.01	0.906	0.01
*v_c_* · *a_p_*	1	152	152	0.05	0.818	0.03
*f* · *a_p_*	1	5058	5058	1.78	0.190	0.085
Error	37	104,904	2835			17.64
Total	46	594,574				

**Table 4:** ANOVA results for cutting parameters influence in passive forces (*F_x_*).

Source	DF	Adj SS	Adj MS	F Value	*p* Value	Impact (%)
Regression model	9	7,819,207	868,801	17.07	0.000	
*v_c_*	1	5,153,459	5,153,459	101.25	0.000	53.08
*f*	1	403,331	403,331	7.92	0.008	4.16
*a_p_*	1	1,922,010	1,922,010	37.76	0.000	19.81
*v_c_* ^2^	1	43,611	43,611	0.86	0.361	0.45
*f* ^2^	1	4054	4054	0.08	0.779	0.04
*a_p_* ^2^	1	9242	9242	0.18	0.672	0.10
*v_c_* · *f*	1	122,529	122,529	2.41	0.129	1.26
*v_c_* · *a_p_*	1	530,247	530,247	10.42	0.003	5.47
*f* · *a_p_*	1	89,582	89,582	1.76	0.193	0.92
Error	37	1,883,330	50,901			19.41
Total	46	9,702,537				

**Table 5:** ANOVA results for cutting parameters’ influence on cutting forces (*F_y_*).

Source	DF	Adj SS	Adj MS	F Value	*p* Value	Impact (%)
Regression model	9	3,370,275	374,475	263.90	0.000	
*v_c_*	1	11,953	11,953	8.42	0.006	0.35
*f*	1	647,708	647,708	456.45	0.000	18.93
*a_p_*	1	2,557,759	2,557,759	1802.51	0.000	74.73
*v_c_* ^2^	1	741	741	0.52	0.475	0.02
*f* ^2^	1	1236	1236	0.87	0.357	0.04
*a_p_* ^2^	1	8524	8524	6.01	0.019	0.25
*v_c_* · *f*	1	538	538	0.38	0.542	0.02
*v_c_* · *a_p_*	1	4355	4355	3.07	0.088	0.13
*f* · *a_p_*	1	278,338	278,338	196.15	0.000	8.13
Error	37	52,503	1419			1.53
Total	46	3,422,778				

**Table 6:** ANOVA results for cutting parameters’ influence on feed forces (*F_z_*).

Source	DF	Adj SS	Adj MS	F Value	*p* Value	Impact (%)
Regression model	9	13,269,293	1,474,366	35.00	0.000	
*v_c_*	1	4,010,562	4,010,562	95.22	0.000	30.23
*f*	1	778,546	778,546	18.48	0.000	5.87
*a_p_*	1	6,485,110	6,485,110	153.97	0.000	4887
*v_c_* ^2^	1	156,218	156,218	3.71	0.062	1.18
*f* ^2^	1	9480	9480	0.23	0.638	0.07
*a_p_* ^2^	1	59,643	59,643	1.42	0.242	0.45
*v_c_* · *f*	1	118,420	118,420	2.81	0.102	0.89
*v_c_* · *a_p_*	1	1,655,970	1,655,970	39.32	0.000	12.48
*f* · *a_p_*	1	709,392	709,392	16.84	0.000	5.35
Error	37	1,558,450	42,120			11.74
Total	46					

**Table 7:** ANOVA results for cutting parameters influence on resultant forces (R).

Source	DF	Adj SS	Adj MS	F Value	*p* Value	Impact (%)
Regression model	9	21,286,032	2,364,115	32.85	0.000	
*v_c_*	1	7,722,548	7,722,548	107.26	0.000	32.24
*f*	1	1,949,865	1,949,865	27.08	0.000	8.14
*a_p_*	1	10,379,067	10,379,067	144.16	0.000	43.35
*v_c_* ^2^	1	245,877	245,877	3.42	0.073	1.03
*f* ^2^	1	15,970	15,970	0.22	0.640	0.07
*a_p_* ^2^	1	40,302	40,302	0.56	0.459	0.17
*v_c_* · *f*	1	124,269	124,269	1.73	0.197	0.52
*v_c_* · *a_p_*	1	1,352,062	4,352,062	18.78	0.000	5.64
*f* · *a_p_*	1	981,866	981,866	13.64	0.001	4.10
Error	37	2,663,962	71,999			11.12
Total	46	23,949,993				

**Table 8:** ANOVA results for cutting parameters’ influence on active power (*P*).

Source	DF	Adj SS	Adj MS	F Value	*p* Value	Impact (%)
Regression model	9	6,853,754	761,528	64.65	0.000	
*v_c_*	1	2,609,862	2,609,862	221.56	0.000	35.79
*f*	1	453,133	453,133	38.47	0.000	6.22
*a_p_*	1	3,629,001	3,629,001	308.07	0.000	49.78
*v_c_* ^2^	1	637	637	0.05	0.817	0.01
*f* ^2^	1	31,828	31,828	2.70	0.109	0.44
*a_p_* ^2^	1	24,953	24,953	2.12	0.154	0.34
*v_c_* · *f*	1	1146	1146	0.10	0.757	0.02
*v_c_* · *a_p_*	1	113,749	113,749	9.66	0.004	1.56
*f* · *a_p_*	1	75,450	75,450	6.41	0.016	1.04
Error	37	435,851	11,780			5.98
Total	46	7,289,604				

**Table 9:** ANOVA results for cutting parameters’ influence on active energy consumption (*E*).

Source	DF	Adj SS	Adj MS	F Value	*p* Value	Impact (%)
Regression model	9	11,126.0	1125.11	317.04	0.000	
*v_c_*	1	2763.7	2763.65	778.75	0.000	26.95
*f*	1	6496.4	6494.41	1830.01	0.000	63.36
*a_p_*	1	41.0	41.03	11.56	0.002	0.40
*v_c_* ^2^	1	111.7	111.74	31.49	0.000	1.09
*f* ^2^	1	395.3	395.32	111.39	0.000	3.85
*a_p_* ^2^	1	1.6	1.65	0.46	0.500	0.02
*v_c_* · *f*	1	217.7	217.72	61.35	0.000	2.12
*v_c_* · *a_p_*	1	1.0	0.97	0.27	0.604	0.01
*f* · *a_p_*	1	0.0	0.03	0.01	0.926	0.00
Error	37	131.3	3.55			1.28
Total	46	10,257.3				

**Table 10:** GRC values for experimental results obtained in the machining process.

*v_c_* (m/min)	*f* (mm/rev)	*a_p_* (mm)	*T* (°C)	*F_x_* (N)	*F_y_* (N)	*F_z_* (N)	*R* (N)	*P* (W)	*E* (W·s)	GRC	Rank
45	0.10	0.50	325.15	240.83	133.06	132.02	305.18	2296.646	72.737	0.91	1
45	0.10	1.25	406.88	284.53	268.39	237.51	457.61	2588.486	77.653	0.76	10
45	0.10	2.10	538.14	382.09	494.49	441.34	765.05	2921.014	79.544	0.62	25
45	0.15	0.50	350.95	413.75	166.36	190.55	484.95	2547.945	53.801	0.81	6
45	0.15	1.25	439.35	402.57	370.51	315.58	631.61	2567.993	52.118	0.71	15
45	0.15	2.10	522.73	418.74	633.43	504.43	911.60	2950.665	53.144	0.61	27
45	0.20	0.50	349.34	312.09	227.00	208.14	438.46	2562.566	41.294	0.83	4
45	0.20	1.25	403.64	297.01	448.45	274.28	603.78	2699.466	40.349	0.74	11
45	0.20	2.10	459.09	313.63	796.02	455.57	969.31	3098.793	43.048	0.63	24
45	0.25	0.50	382.80	350.49	224.03	155.80	444.19	2620.315	34.937	0.82	5
45	0.25	1.25	428.96	412.01	584.93	356.61	799.42	2771.530	33.879	0.69	20
45	0.25	2.10	505.12	314.25	1043.46	540.13	1216.26	3161.184	34.260	0.60	28
70	0.10	0.50	416.50	333.37	141.23	156.35	394.37	2544.727	53.028	0.81	7
70	0.10	1.25	461.61	405.66	287.29	317.65	589.91	2860.079	55.618	0.70	19
70	0.10	2.10	584.47	930.21	495.59	644.76	1235.56	3211.256	57.107	0.53	35
70	0.15	0.50	426.40	518.99	180.61	226.87	594.51	2898.521	39.336	0.74	12
70	0.15	1.25	550.16	962.57	402.21	620.98	1214.05	3049.665	38.969	0.57	30
70	0.15	2.10	525.27	1218.20	652.19	1094.42	1762.70	3438.348	40.108	0.49	42
70	0.20	0.50	361.25	273.66	211.17	143.16	374.14	2759.718	27.896	0.86	2
70	0.20	1.25	458.42	515.67	477.89	379.16	798.79	3038.281	29.529	0.67	21
70	0.20	2.10	497.86	811.75	793.96	756.86	1364.61	3595.598	32.949	0.54	34
70	0.25	0.50	350.33	444.02	227.65	192.37	534.78	2747.419	22.899	0.83	3
70	0.25	1.25	471.39	944.92	545.52	641.95	1265.92	3100.075	24.108	0.60	29
70	0.25	2.10	637.55	1765.36	1213.79	2179.85	3056.39	3881.642	28.042	0.40	47
85	0.10	0.50	389.57	428.03	173.10	224.18	513.25	2866.254	50.149	0.76	9
85	0.10	1.25	508.07	561.27	294.74	440.76	772.12	3042.898	48.201	0.64	23
85	0.10	2.10	563.04	1307.91	472.20	945.03	1681.27	3284.633	47.446	0.50	41
85	0.15	0.50	443.69	750.47	206.40	289.73	830.51	2894.179	33.764	0.70	18
85	0.15	1.25	532.20	1219.17	401.22	892.23	1563.15	3191.112	34.560	0.54	33
85	0.15	2.10	533.61	1079.66	647.78	1133.06	1693.85	3425.260	33.281	0.50	39
85	0.20	0.50	432.24	619.57	224.00	262.71	709.26	2954.654	26.262	0.73	13
85	0.20	1.25	481.15	821.72	470.16	644.97	1145.53	3251.394	25.293	0.61	26
85	0.20	2.10	527.27	941.75	836.46	1344.99	1842.70	3619.628	26.133	0.50	40
85	0.25	0.50	393.55	608.74	213.76	231.45	685.44	2937.999	20.391	0.78	8
85	0.25	1.25	545.01	1580.97	595.64	1122.52	2028.38	3383.327	21.623	0.52	37
85	0.25	2.10	563.09	1618.52	1079.50	2118.15	2876.02	3601.041	21.006	0.45	44
95	0.10	0.50	500.44	855.56	154.81	383.80	950.39	2924.417	44.429	0.66	22
95	0.10	1.25	550.61	1004.05	316.89	734.15	1283.55	3218.591	46.478	0.55	31
95	0.10	2.10	614.38	1146.54	553.52	1477.54	1950.40	3596.735	47.969	0.45	43
95	0.15	0.50	435.06	880.84	172.39	355.25	965.30	2854.485	28.826	0.70	17
95	0.15	1.25	595.84	1402.56	449.52	1134.40	1859.06	3308.830	31.260	0.51	38
95	0.15	2.10	600.86	1735.54	774.05	2077.89	2815.83	3983.394	36.508	0.40	46
95	0.20	0.50	412.06	750.95	205.02	326.08	843.97	2985.273	23.214	0.73	14
95	0.20	1.25	573.37	1335.06	531.62	1120.10	1821.99	3425.553	23.798	0.52	36
95	0.20	2.10	615.84	1789.92	903.21	2088.30	2894.92	4077.910	26.051	0.42	45
95	0.25	0.50	431.55	1021.14	215.65	362.78	1104.92	2925.051	17.875	0.71	16
95	0.25	1.25	507.96	1320.04	591.83	1077.33	1803.72	3444.203	19.147	0.55	32

**Table 11:** Optimization results for the mathematical models obtained.

Rank	*v_c_* (m/min)	*f* (mm/rev)	*a_p_* (mm)	*T* (°C)	*F_x_* (N)	*F_y_* (N)	*F_z_* (N)	*R* (N)	*P* (W)	*E* (W·s)	GRC
1	45	0.25	0.5	342.88	235.15	232.42	90.71	357.04	2543.340	33.441	0.9036
2	45	0.24	0.5	341.47	235.20	224.54	99.03	354.40	2547.793	34.487	0.9031
3	45	0.23	0.5	340.31	236.00	217.06	108.49	353.24	2550.157	35.767	0.9018
4	46	0.25	0.5	344.07	244.11	231.67	87.12	358.10	2553.160	32.888	0.9016
5	46	0.24	0.5	342.70	243.67	223.82	94.95	354.96	2557.662	33.913	0.9012
6	46	0.23	0.5	341.57	243.97	216.38	103.93	353.30	2560.073	35.172	0.9000
7	45	0.22	0.5	339.38	237.55	210.01	119.10	353.56	2550.429	37.279	0.8999
8	47	0.25	0.5	345.28	253.30	230.95	83.96	359.71	2562.952	32.346	0.8995
9	47	0.24	0.5	343.95	252.36	223.13	91.31	356.07	2567.503	33.351	0.8992
10	46	0.22	0.5	340.69	245.02	209.36	114.04	353.13	2560.393	36.663	0.8981

**Table 12:** Optimization results for the energy consumption adequation.

Rank	*v_c_* (m/min)	*f* (mm/rev)	*a_p_* (mm)	*T* (°C)	*R* (N)	*E’* (W·s)	GRC
1	45	0.24	0.5	341.48	354.40	144.857	0.8381
2	45	0.23	0.5	340.31	353.24	150.220	0.8376
3	45	0.25	0.5	342.88	357.04	140.452	0.8373
4	46	0.24	0.5	342.70	354.96	142.435	0.8368
5	46	0.23	0.5	341.58	353.30	147.721	0.8362
6	46	0.25	0.5	344.07	358.10	138.129	0.8360
7	45	0.22	0.5	339.38	353.56	156.571	0.8356
8	47	0.24	0.5	343.95	356.07	140.073	0.8354
9	47	0.23	0.5	342.87	353.91	145.270	0.8347
10	47	0.25	0.5	345.28	359.71	135.854	0.8346

## Data Availability

The original contributions presented in this study are included in the article. Further inquiries can be directed to the corresponding author.
